# Development of a Web-Based Survey for Monitoring Daily Health and its Application in an Epidemiological Survey

**DOI:** 10.2196/jmir.1872

**Published:** 2011-09-23

**Authors:** Hiroaki Sugiura, Yasushi Ohkusa, Manabu Akahane, Tomomi Sano, Nobuhiko Okabe, Tomoaki Imamura

**Affiliations:** ^1^Department of Public Health, Health Management and PolicyNara Medical University School of MedicineKashiharaJapan; ^2^Infectious Disease Surveillance CenterNational Institute of Infectious DiseaseTokyoJapan

**Keywords:** Web-based survey, syndromic surveillance, long-term operation

## Abstract

**Background:**

Early detection of symptoms arising from exposure to pathogens, harmful substances, or environmental changes is required for timely intervention. The administration of Web-based questionnaires is a potential method for collecting information from a sample population.

**Objective:**

The objective of our study was to develop a Web-based daily questionnaire for health (WDQH) for symptomatic surveillance.

**Methods:**

We adopted two different survey methods to develop the WDQH: an Internet panel survey, which included participants already registered with an Internet survey company, and the Tokyo Consumers’ Co-operative Union (TCCU) Internet survey, in cooperation with the Japanese Consumers’ Co-operative Union, which recruited participants by website advertising. The Internet panel survey participants were given a fee every day for providing answers, and the survey was repeated twice with modified surveys and collection methods: Internet Panel Survey I was conducted every day, and Internet Panel Survey II was conducted every 3 days to reduce costs. We examined whether the survey remained valid by reporting health conditions on day 1 over a 3-day period, and whether the response rate would vary among groups with different incentives. In the TCCU survey, participants were given a fee only for initially registering, and health information was provided in return for survey completion. The WDQH included the demographic details of participants and prompted them to answer questions about the presence of various symptoms by email. Health information collected by the WDQH was then used for the syndromic surveillance of infection.

**Results:**

Response rates averaged 47.3% for Internet Panel Survey I, 42.7% for Internet Panel Survey II, and 40.1% for the TCCU survey. During a seasonal influenza epidemic, the WDQH detected a rapid increase in the number of participants with fever through the early aberration reporting system.

**Conclusions:**

We developed a health observation method based on self-reporting by participants via the Internet. We validated the usefulness of the WDQH by its practical use in syndromic surveillance.

## Introduction

The collection of health crisis information has been an important task in every country since the 2005 implementation of the World Health Organization’s International Health Regulations to prevent the global spread of illness [1]. Early detection of health events related to exposure to various pathogens, harmful substances, or environmental changes is indispensable for timely intervention to minimize health crises.

Syndromic surveillance is a method used to investigate epidemics of infections [2-5]. Unlike sentinel surveillance, which uses a traditional definitive diagnosis and pathogen identification, this method encompasses the surveillance of symptoms. For example, this type of surveillance has been used at medical institutions to determine the number of patients with fever, cough, diarrhea, or vomiting, and changes in the number of absentees from school or the workplace, sales of commercial drugs, and prescriptions [6-8]. Syndromic surveillance is important as a means of gathering information during the early stages of an epidemic, and it has practical application in many countries. Thus, an effective means of collecting daily health information from people directly and quickly is desirable.

Use of the Web to perform an epidemiological survey was reported in 1996 [9]. This method has since been applied to national-scale surveys in various countries where residents voluntarily input information on influenza-like symptoms directly into a dedicated website so that epidemiologists can gain an understanding of the influenza epidemic [10-13].

To broaden the range of such a survey in terms of contributors and infectious diseases, we developed and conducted a daily health survey of the general population using the Internet and named this survey the Web-based daily questionnaire for health (WDQH) [14]. We report the methodology of data collection and processing of the WDQH and clarify its use in syndromic surveillance. We performed this study with participants in panels registered at Internet survey companies. In addition, we investigated a method for the long-term operation of the survey by reducing the cost of each individual survey.

## Methods

### Recruitment

Two different methods were used to recruit participants for the WDQH. First, Internet panel surveys comprised people who were already registered with an established Internet survey company. Second, the Tokyo Consumers’ Co-operative Union (TCCU) Internet survey comprised members of the TCCU, in cooperation with the Japanese Consumers’ Co-operative Union (JCCU), who were invited to participate via advertising on the company website.

An Internet survey company conducts questionnaire surveys via the Internet. For survey participants registered in advance, questionnaires and a response column are displayed on the website for the respondents to complete and transmit their responses. Additionally, the Internet panel survey was repeated twice with different survey and collection methods (Internet panel surveys I and II). In the Internet panel surveys, the respondents were registered as panel members with the company and were residents of Izumo City (150,000 inhabitants) in western Japan, which had 89.5% Internet coverage in 2008. The youngest respondent was 16 years old. The respondents also provided information regarding symptoms in family members. Internet Panel Survey I was conducted daily between December 1, 2007, and March 28, 2008, among 245 respondents who were paid 60 yen (US $0.75;US $1.00 = 80 yen at the time of writing) per survey completed. A reminder email was sent daily to those who agreed to participate. Internet Panel Survey I included 702 registrants. Respondents were those who completed the survey, and all family members included in the survey were considered to be participants.

**Figure 1:**
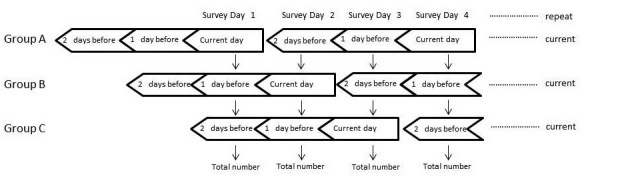
Data collection method for Internet Panel Survey II.

In Internet Panel Survey II, conducted between January 8 and March 13, 2009, we examined changes in the data acquisition method to reduce survey costs. We investigated whether reporting health conditions once every 3 days could reduce survey costs. Internet Panel Survey II was conducted continuously with 264 respondents and included 716 registrants. The respondents were divided into groups A, B, and C, and each group was surveyed by shifting the survey date by 1 day to determine the applicability of recall for 1-in-3-day reporting. Thus, groups A, B, and C received the questionnaire on days 1, 2, and 3, respectively. For example, the data on survey day 1 included symptoms that were experienced on the current day by group A, on the day before the survey day by group B, and 2 days before the survey day by group C. Thus, on survey day 1, responses were obtained from all the respondents (Figure 1). Each group was divided randomly. A reminder email was sent to each group on the survey day.

We also investigated whether the response rate varied according to the incentive. Each group was further divided into three subgroups in which the members were given a reward of 40 (US $0.50), 60 (US $0.75), or 80 yen (US $1.00). The response rates were then investigated.

In the TCCU survey, we examined methods of collecting health information from the city’s residents without the use of an Internet survey company. The respondents were those who accessed the website of the TCCU’s home delivery services and applied to participate in the survey, which was advertised with an onscreen banner. Participants were recruited between January 15 and January 31, 2009, and any applicant could participate. There were 427 respondents from Tokyo, which had 95.2% Internet coverage in 2008. They were given 100 yen (US $1.25) for registering. No fee was paid for each survey, but health information was provided to the participants in the relevant residential areas based on survey results. The TCCU survey was conducted in cooperation with the JCCU, which has 1 million members in Tokyo among a population of 12.3 million. The TCCU has a strong corporate philosophy regarding food safety and understanding the health concerns of consumers. Many respondents were homemakers, as the proportion of female respondents was 97.6%. They provided information about themselves and family members, providing 1453 participants, who were 49.5% male and 50.5% female.

### Response Method

On the day of the survey, the survey administrator sent a reminder email to all those recruited. Respondents accessed the password-protected website designated in the email and responded to the questions. The questionnaire ascertained whether respondents or their family members had any symptoms. The gender and age (in 5-year intervals) of those who developed symptoms as well as their specific symptoms (Table 1) were noted. In Internet Panel Survey I, 6 symptoms associated with diseases of infection and bioterrorism were selected. In Internet Panel Survey II and in the TCCU survey, 12 symptoms associated with seasonal allergic diseases and changes in body conditions were added, and “fever” was divided into “slight fever” and “high fever.”

**Table 1:** Items in the three surveys

Internet Panel Survey I	Internet Panel Survey II (TCCU^a^ survey)
Fever	Slight fever
Cough	High fever
Diarrhea	Runny nose
Vomiting	Cough
Eruption	Diarrhea
Convulsion	Vomiting
	Convulsion
	Eye itch
	Eruption
	Diagnosis of influenza
	Diagnosis of gastroenteritis
	Arthritic pain
	Muscle pain
	Shoulder stiffness
	Sneeze
	Skin itch
	Rough hands
	Sleeplessness
	Decreased concentration

^a^ Tokyo Consumers’ Co-operative Union.

These surveys were conducted with varied symptoms to examine whether the WDQH could be applied in the surveillance of various diseases according to symptoms.

**Figure 2:**
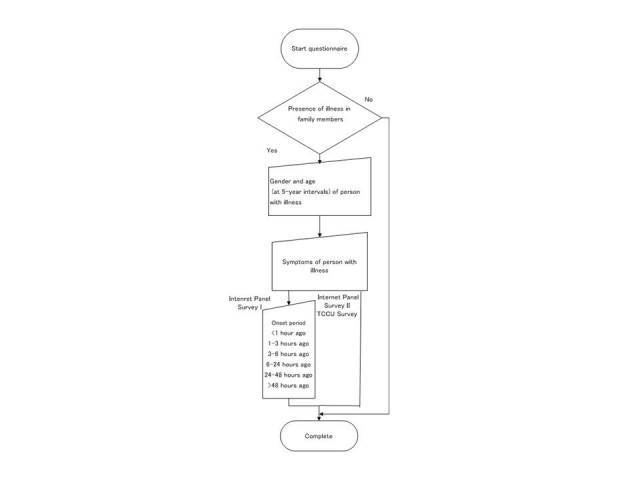
Flowchart for all three surveys (TCCU = Tokyo Consumers’ Co-operative Union).

Reports by asymptomatic people are essential for calculating prevalence rates and an analysis of variance by the presence of symptoms. The symptoms quoted in this survey are common, particularly during the acute phases of diseases caused by infection and environmental factors. The time of symptom onset was determined (<1 hour ago, 1–3 hours ago, 3–6 hours ago, 6–24 hours ago, 24–48 hours ago, and >48 hours ago) (Figure 2) in Internet Panel Survey I.

In Internet Panel Survey II and the TCCU survey, only cases with an onset on the current day were reported. If a respondent tried to exit the survey without answering all the questions, the system would alert the respondent in order to prevent invalid responses.

### Data Processing

**Figure 3:**
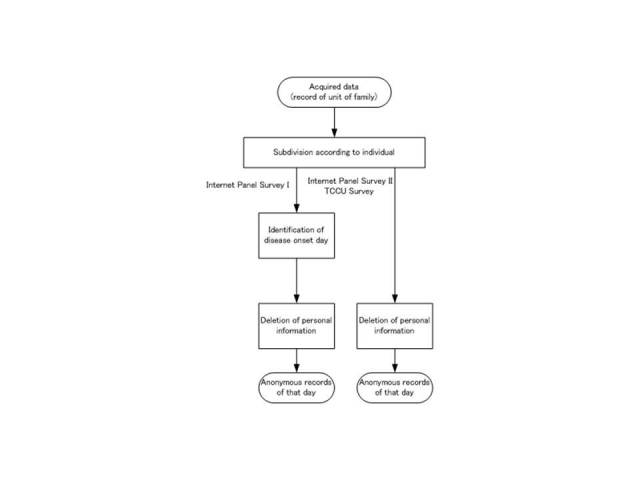
Data processing flowchart for all three surveys (TCCU = Tokyo Consumers’ Co-operative Union).

The survey also included details such as survey date, presence of illness in family members, presence of symptoms in family members, and time of symptom onset in a family member. Data entered in the WDQH were then transmitted to a server managed by a researcher. Subsequently, the records for each household were subdivided by family member. In Internet Panel Survey I, the symptom onset dates for participants were determined from the time elapsed between symptom onset and reporting. Those with a symptom onset >48 hours before the survey were excluded. This was intended to include only people with symptoms at an acute stage. Personal information was then deleted (Figure 3).

The final participant records consisted of survey date, presence of illness and presence of symptoms by participant, and symptom onset date. Cross-correlation was used during syndromic surveillance with the collected data to determine the number of participants by symptom and date.

### Examples of Using the Data in Syndromic Surveillance

Symptoms were cross-tabulated to determine the symptom onset dates and number of participants who developed a particular symptom. The results were used to prepare time-series graphs by symptom, with the prevalence of symptoms plotted against the date. Subsequently, alerts by symptom were reported on the day when the number of participants who developed the symptom increased rapidly compared with the baseline of the previous 10 days using the early aberration reporting system (EARS) algorithm recommended by the US Centers for Disease Control and Prevention [15,16].

This study was approved by the Ethical Committee of Nara Medical University (Authorization Code: 220).

## Results

### Respondents’ Demographic Characteristics and Response Rates

Table 2 presents the number of respondents, gender, age distribution, number of participants including families of the respondents, and daily mean response rates for the three surveys. The numbers of respondents (total number of participants) in Internet Panel Survey I, Internet Panel Survey II, and the TCCU survey were 245 (702), 264 (716), and 427 (1453), respectively.

**Table 2:** Demographics and response rates of participants in the three surveys

		Internet Panel Survey I	Internet Panel Survey II	TCCU^a^ survey
**Number of respondents**		245	264	427
	Men	44.5%	52.7%	2.6%
	Women	55.5%	47.3%	97.6%
**Age distribution of respondents (years)**				
	≤29	26.5%	26.9%	4.6%
	30–39	43.3%	41.7%	35.3%
	40–49	21.9%	21.9%	39.4%
	50–59	5.8%	7.9%	16.3%
	≥60	2.5%	1.6%	4.4%
Daily mean response rate		47.3%	42.7%	40.1%
Total number of participants		702	716	1453

^a^ Tokyo Consumers’ Co-operative Union.

**Figure 4:**
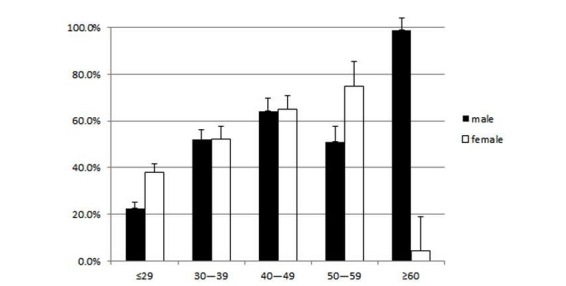
Population distribution by age in the surveys. Data are presented as the mean and standard deviation, which is indicated by error bars. Age is given in years.

In Internet Panel Survey I, a constant response rate was observed from the initiation to the end of the survey, and there was no tendency to respond when only 1 symptom was present. The response rate was 48.7% on weekdays and 44.4% on the weekend, indicating a significantly higher rate on weekdays (*P* < .001). In addition, the percentage of respondents with a 100% response rate was 3.2%, whereas the percentage of those with no responses was 34.5%. The response of “presence of fever” was given by 184 participants, including family members of the respondent. Among these, data for 2 participants were given for the first time only when the symptom was present. The daily mean response rate was determined for different ages and genders. When those aged ≥60 years were excluded from the analysis, the lowest response rate was 22.6% for males aged ≤29 years, and the highest response rate was 74.9% for women aged 50–59 years (Figure 4).

In Internet Panel Survey II, the response rate was constant from the initiation of the survey to its end. The response rate was 44.0% on weekdays and 39.9% on the weekend. The percentage of respondents with a 100% response rate was 6%, and the percentage of those without a response was 36.2%.

In the TCCU survey, the response rate decreased gradually from the first to the final day. The response rate was 41.5% on weekdays and 38.9% on weekends. No significant differences were observed among the groups. The percentage of respondents with a 100% response rate was 3.3%, and the percentage of those without a response was 5.9%.

### Elapsed Time From the Development of Symptoms to a Report

In Internet Panel Survey I, the appearance of symptoms was as follows: >48 hours ago (59%), 6–24 hours ago (13%), 24–48 hours ago (12%), 3–6 hours ago (3%), 1–3 hours ago (1%), and <1 hour ago (1%). Of all responses, the daily average reporting rates by symptom were as follows: cough (8%), fever (3%), diarrhea (2%), vomiting (1%), rash (0%), and convulsion (0%).

### Examples of Using the Data in Syndromic Surveillance

**Figure 5:**
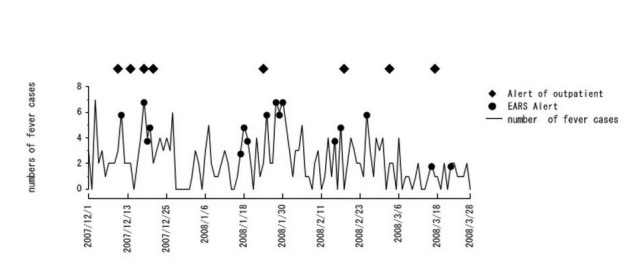
Results of syndromic surveillance conducted in Internet Panel Survey I. Circles: alerts reported by early aberration reporting system (EARS). Diamonds: alert occurrence dates coincident with the regional outpatient symptomatic surveillance in medical institutions.

Figure 5 presents a graph for fever in Internet Panel Survey I.

The number of participants with fever was made a parameter. When the number of persons with fever was 3 or more standard deviations above the mean of the previous week, EARS provided an alert [15]. The number of those who reported febrile symptoms at history-taking in the outpatient section was made a parameter. The outpatient symptomatic surveillance reported 8 alerts, whereas the WDQH reported 16 alerts. Because of the time factor in an epidemic, the presence of an alert within the gold standard (3 days before to 3 days after symptom onset) was examined. The sensitivity was 0.43, and the specificity was 0.88. For cough, 8 alerts were reported during outpatient symptomatic surveillance. In Internet Panel Survey I, 19 alerts were reported. Similarly, for diarrhea, there were 30 alerts in outpatient symptomatic surveillance and 25 alerts in Internet Panel Survey I. For vomiting, there were 24 alerts in outpatient symptomatic surveillance and 22 alerts in Internet Panel Survey I. For rash, there was 1 alert in outpatient symptomatic surveillance and 10 alerts in Internet Panel Survey I. For convulsions, there was 1 alert in outpatient symptomatic surveillance and 7 alerts in Internet Panel Survey I.

**Figure 6:**
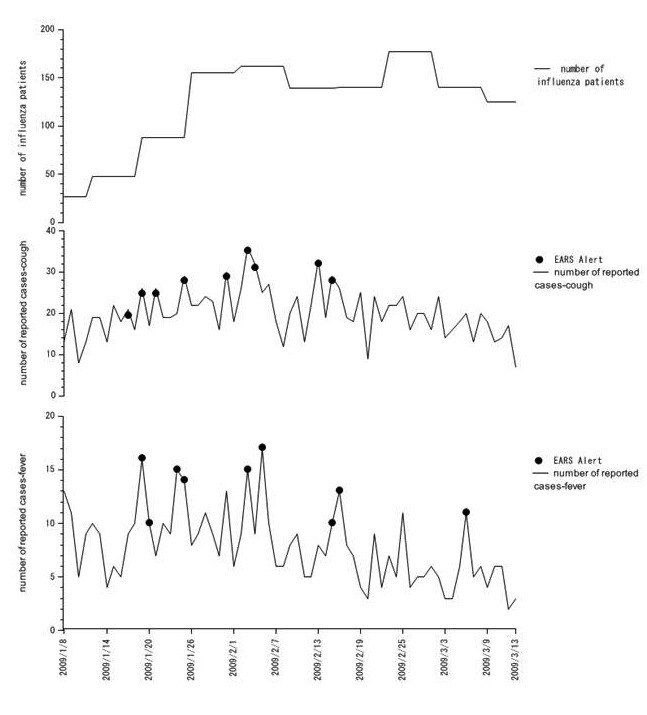
Results of syndromic surveillance conducted in Internet Panel Survey II. Circles: alerts reported by early aberration reporting system (EARS).

Figure 6 shows the results of syndromic surveillance in Internet Panel Survey II: the number of participants with influenza in the area (published by the trend of symptom onset), those who reported cough, and those with fever. During the survey, 9 alerts each were reported for cough and fever.

### Examination of Methods to Reduce Survey Costs

Table 3 shows the fixed, variable, and total costs for the three surveys. Initial costs were very low only for screening the questions for panel research. Variable costs consisted of the investigation days and the number of investigation panels. In the TCCU survey, the fixed costs for development were the highest. Variable costs were only for incentives paid when participation was declared.

**Table 3:** Survey expenses for the three surveys

		Internet Panel Survey I	Internet Panel Survey II	TCCU^a^ survey
**Fixed cost**				
	Yen	20,000	20,000	2,457,000
	US $	250	250	30,712.5
**Variable cost**				
	Yen	8,260,000	2,480,000	43,000
	US $	103,250	31.000	537.5
**Total cost**				
	Yen	8,280,000	2,500,000	2,500,000
	US $	103,500	31,250	31,250

^a^ Tokyo Consumers’ Co-operative Union.

There were 3 respondents in Internet Panel Survey II. All respondents gave their answers regarding symptoms that presented on the same day, providing responses on the day, the day after, and 2 days after the sentinel day. The response rates were 42.4%, 43.1%, and 42.7% in groups A, B, and C, respectively. No significant difference was found in the response rate among the groups.

The response rates by fee paid for a single response were as follows: 46.7% (40 yen), 39.7% (60 yen), and 41.6% (80 yen). A 1-way analysis of variance revealed a significant difference; thus, a multiple comparison test was conducted. Significant differences were observed in the average response rates between the 40-yen and 60-yen groups and between the 40-yen and 80-yen groups, with a greater response rate in the 40-yen group.

## Discussion

We developed and validated a health observation method based on self-reporting by participants via the Internet. We clarified the usefulness of the WDQH by its practical use for syndromic surveillance.

Conventional paper-based surveys can be conducted at a low cost in a small population, and these surveys do not incur major initial expenses for the system. Moreover, combining Web- and paper-based surveys improves the response rate [17]. However, although requiring greater up-front costs, the WDQH allows daily inexpensive repetitive surveys to be conducted in a large number of participants, illustrating the advantage of a Web-based survey [18]. Furthermore, Web-based surveys permit a more efficient statistical analysis of data by computer. Thus, cost-effective and rapid surveys of a large number of participants, with high data precision, have become possible.

Previous studies have reported Internet surveys of asthma and diet, for example, in specific groups and patients [19,20]. Various countries have been using a method to understand an influenza epidemic in which residents voluntarily input information on influenza-like symptoms directly into a website. Thus, this method has been verified with an actual influenza epidemic and its usefulness has been demonstrated [10,13].

Although reports are available on the surveillance of symptoms in volunteers, no reports are available on the surveillance of symptoms of people identified by an Internet survey company. To promote the robustness of data gathering, the WDQH was conducted among registered members of an Internet survey company who were more likely than anonymous respondents to provide reliable data. Additionally, because the respondents were recruited from among registered members, only a short time was required from the decision making at the initiation of the survey to actual data collection. Thus, this survey provides value in this regard.

Rates of 52.6% [21] and 50% [22] have been reported in surveys that ended after a single investigation. In a meta-analysis conducted on 68 response rates of sampling surveys, the average response rate for Web-based surveys was 39.6% [23]. During surveillance of symptoms in volunteers, some participants who initially did not respond to the survey responded only when a symptom was present [13]. In our study, respondents to the Internet panel surveys who completed the questionnaire the first time tended to always cooperate with the survey. This finding indicates that these surveys are a useful method for reporting the appearance of symptoms.

There are problems with previous surveillance methods, such as the length of time required, indirect data collection, and no data collection during holidays. However, we developed the WDQH with the objective of acquiring data immediately after symptom onset. In addition, the WDQH allowed data collection on Saturdays, Sundays, and public holidays. Thus, we were able to conduct consistent daily surveillance. Furthermore, we used preventive measures, such as a branched and stepwise-structured questionnaire, to eliminate mistakes and discrepancies in responses [24].

Internet Panel Survey I confirmed that participants’ health information could be collected daily via the Internet. However, the survey cost was 8.28 million yen (US $103,500), which was considered too expensive over a long period. We thus conducted both Internet Panel Survey II, which is economical for a panel survey, and the TCCU survey, without using the Internet survey company. In Internet Panel Survey II, two surveys were conducted. The first was used to reduce the frequency of surveys to once every 3 days. Changes in the actual number of participants with influenza corresponded with the changes determined by Internet Panel Survey II, which was conducted for syndromic surveillance without impairing data precision. This method allowed the implementation of a survey 3 times as long for the same cost as one conducted daily. The cost of Internet Panel Survey II was approximately one-half that of Internet Panel Survey I.

The second survey in Internet Panel Survey II investigated cash incentives. The Internet survey company that we used typically paid a fee of 60 yen for a single response. Surprisingly, the response rate was highest when the fee was set at the lowest level of 40 yen. Generally, higher fees act as an incentive for recruitment, but this study found that the offer of a higher reward did not result in a higher response rate. This point has been supported by a previous study [25]. As there was a sufficient response with no payment for each TCCU survey, any cost-associated restrictions on the survey period were eliminated. A fee was paid to the members of the TCCU only for survey registration, and information about the results was provided to the respondents. The response rate for the TCCU survey was lower than that for Internet panel surveys I and II. However, even at this lower rate, a large number of members were included because membership in the JCCU numbers at least 24 million throughout Japan, including 300,000 registered to its website.

From the WDQH data, we used EARS as an alert so that a level measured on the current day that was greater than 3 standard deviations different from the mean observed level for the previous week was reported as abnormal. If data are accumulated for several years, the number of participants can be estimated by multivariate analyses, where the number of participants, number of weeks, day of the week, holidays, and day after holidays are considered dummy variables. However, in this study, data were not continuously accumulated for 1 year or longer; thus, a multivariate analysis was not performed.

Syndromic surveillance could be implemented as a result of these validations. The Internet survey company in this study used an existing survey panel. The time required from planning to implementation was short with the use of this company, which already had its registered members as recruited participants. Thus, an urgent surveillance can be conducted within 3 days regardless of the location in Japan. During syndromic surveillance using the WDQH, measures against a health crisis can be readily put in place.

Removing selection bias is difficult in Internet surveys. The population tended to be biased toward young people because Internet surveys require respondents to have computer skills. Introducing an easy system to increase the response rate of older people could reduce this bias. However, because we believe that the increase or decrease in symptoms is reliable regardless of bias, we used EARS for all methods. Cough was often excluded from previous surveys because most cases of cough were present >48 hours before the survey, and cough probably requires a longer time to be recognized as bothersome to the same degree as other symptoms, such as fever and vomiting. To use the WDQH for syndromic surveillance, questions to respondents should be limited to those regarding acute symptoms, and a system that allows easy reporting within 24 hours should be established in the future.

In this study, we conducted the surveys with the same respondents. We think that it would be difficult for the respondents to maintain their interest every day for several years. For long-term operation of the survey, we consider that new respondents should be recruited after a certain period.

There are two further applications for the surveys other than the surveillance of symptoms. First, when environmental data published later by public institutions, such as average temperature and atmospheric pressure, are linked to the records by participant and locality on the same date, a cross-correlation survey of symptoms and environmental factors can be implemented. In the future, various daily surveys can be conducted, such as those for mean air temperature and the presence or absence of fever. These are topics to be investigated in the future.

Another application for the WDQH could be in postmarketing surveillance of food similar to that conducted for pharmaceuticals. Food safety is more widely expected by consumers today than it was in the past. To date, postmarketing surveillance of food has been conducted in only a single instance for a food additive [26]. A cross-correlation survey using a consumer database to identify the relationship between daily symptom data of the respondents obtained by the WDQH and consumed foods based on sales records may allow the reporting of adverse events when certain symptoms are associated with specific products.

### Conclusions

We developed a health observation method via the Internet using self-reporting by respondents and validated the method for its application in syndromic surveillance. The Internet allows quick, cost-effective epidemiological surveys to be conducted that would be difficult to conduct by conventional methods.

## Multimedia Appendix 1

CHERRIES checklist for Internet panel surveys I and II and the Tokyo Consumers’ Co-operative Union (TCCU) survey.

## Abbreviations

EARS: early aberration reporting system
JCCU: Japanese Consumers’ Co-operative Union
TCCU: Tokyo Consumers’ Co-operative Union
WDQH: Web-based daily questionnaire for health
